# In Memoriam—Martin C. Mihm, Jr.

**DOI:** 10.3390/dermatopathology9030036

**Published:** 2022-09-08

**Authors:** Thomas Kupper, Adriano Piris, Daniela Kroshinsky, Gürkan Kaya

**Affiliations:** 1 Department of Dermatology, Brigham and Women’s Hospital, Boston, MA 02115, USA; 2 Department of Dermatology, Massachusetts General Hospital, Boston, MA 02114, USA; 3 Departments of Dermatology and Clinical Pathology, University Hospital of Geneva, 1205 Geneva, Switzerland

Martin C. Mihm, Jr. (15 March 1934–19 July 2022), Professor of Dermatology, Pathology and Dermatopathology, Harvard Medical School, Boston, MA, USA (Figure 1).

Dr. Martin C. Mihm, Jr. passed away on 19 July 2022, and the whole dermatopathology community is deeply saddened by this sudden loss. Dr. Mihm, a world-renowned international consultant for melanoma, other skin tumors and cutaneous inflammatory diseases, was one of the greatest names in dermatology and dermatopathology, and he made countless contributions to these fields.

Dr. Mihm graduated from the University of Pittsburgh, School of Medicine and joined the Massachusetts General Hospital (MGH) at Harvard Medical School as a trainee in Dermatology in 1964. He was board-certified in Dermatology, Pathology and Dermatopathology. However, it was his passion for dermatopathology that led to an appointment as the Chief of the MGH Dermatopathology Unit in 1974, where he served for the next two decades. Within the Department of Dermatology at the Brigham and Women’s Hospital and Dana-Farber and Brigham and Women’s Cancer Center, where he remained the Director of the Mihm Cutaneous Pathology Consultative Service, a second opinion dermatopathology service and the Director of the Melanoma Program, Dr. Mihm consulted on more than 350,000 cases, nationally and worldwide, and offered accurate diagnosis, prognosis and treatment recommendations for routine and challenging cases in dermatology and dermatopathology. He also served as an associate director of the Center for Melanoma Oncology at Dana-Farber and Brigham and Women’s Cancer Center, as a co-director of the Melanoma Risk and Prevention Clinic at Dana-Farber and Brigham and Women’s Cancer Center and as a Professor of Dermatology at Harvard Medical School. Dr. Mihm was also active internationally; he was a co-founder of the Rare Tumor Institute of the WHO in Milan, Italy, and acted as an external coordinator for five years. He was also the co-director of the Melanoma Pathology Program of the European Organization for the Research and Treatment of Cancer (EORTC).

Since the beginning, he was interested in melanoma pathology, and with his mentor and friend, Wallace Clark, he published a paper in 1969 entitled “The histogenesis and biological behavior of primary human malignant melanoma of the skin” [1]. In 1971, Dr. Mihm and Dr. Clark published a seminal paper entitled “The clinical diagnosis, classification and histogenetic concepts of the early stages of cutaneous malignant melanoma” in the *New England Journal of Medicine* [2]. In these articles, he described the different levels of melanoma invasion which were then used worldwide by dermatopathologists.

Dr. Mihm was the first director of the Harvard Dermatopathology Training Program, founded in 1975. His precise and revolutionary approach to the practice of cutaneous pathology led to the birth of modern dermatopathology, and thanks to his inexhaustible academic energy, he trained countless dermatologists and dermatopathologists all over the world. In 2010, a special issue was published in the *Journal of Cutaneous Pathology* to honor Dr. Mihm’s 75th birthday and his achievements [3].

Dr. Mihm loved giving lectures worldwide; he accepted numerous invitations nationally and internationally. He had been in Geneva, Switzerland many times to give his great lectures for clinicopathological meetings and to train dermatopathologists in different courses organized by the University Hospital of Geneva Dermatopathology.

Dr. Mihm published more than 400 original manuscripts, more than 100 reviews and chapters and more than a dozen textbooks and monographs. Among the very long list of his substantial contributions to dermatopathology, we can cite the following: the recognition of melanoma tumor progression: radial and vertical growth; the identification of different clinicopathological types of melanoma; the recognition of immunologic variants of cutaneous necrotizing vasculitis; the identification of syngeneic human graft-versus-host disease; the definition of the specificity and clinical utility of the lupus band test; the discovery of CD1a expression on human Langerhans cells and the demonstration of the utility of CD1a immunophenotyping for Langerhans cell histiocytosis; the recognition of borderline/minimal deviation melanoma variants; the introduction of classification schema for conjunctival melanocytic lesions; the development of a grading system for cytologic atypia in dysplastic nevi; the characterization of nevoid melanoma; the significance of tumor infiltrating lymphocytes in melanoma nodal metastases; the description of osteogenic melanoma; the establishment of guidelines for histological reporting of melanoma excisions; the clinicopathological insights into animal-type melanoma; the description of pigmented epithelioid melanocytoma; the description of use of MITF in melanoma diagnosis; the key contributions to AJCC melanoma staging; the discovery of a new marker (GLUT1) for juvenile hemangiomas; the contribution to the development of engineered melanoma vaccines based on dendritic cells; the setting of an EORTC melanoma policy; the identification of tumor lymphangiogenesis as a novel indicator of melanoma prognosis; the identification of the biology of desmoplastic melanoma; the elucidation of lentiginous subtype melanoma; the discovery that Prox-1 promotes the invasion of kaposiform hemangioendotheliomas [3].

Dr. Mihm was an outstanding physician, scientist and dermatopathologist; however, he also had great humanistic qualities. Despite his huge medical and scientific knowledge, he was humble, eternally optimistic and always wanted to help people around him. He was a hero, a father figure and a spiritual guide for many colleagues. He was a rare intellect, spoke several foreign languages, including German, French and Italian, had a great culinary taste and loved classical music. He always said that if he were not a physician, he would want to become the Chief Conductor of a Symphony Orchestra.

Marty, as we, your close friends call you, wonderful friend, educator, and mentor, you have been the Chief Conductor of Dermatopathology; we have been fortunate to experience your teaching, brilliance, kindness and generosity. Thank you for what you have done for dermatology and pathology. Rest in peace. We will never forget you!

## Figures and Tables

**Figure 1:**
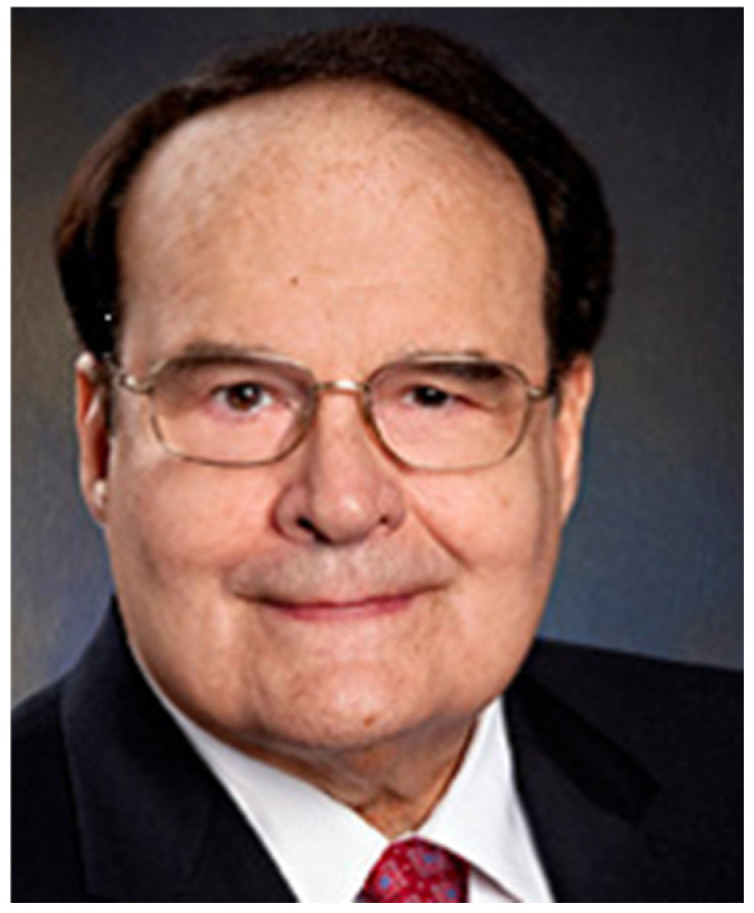
The Photo of Prof. Dr. Martin C. Mihm, Jr.
